# Multiple variables influence the finely calibrated antioxidant defenses of *Clostridioides difficile*

**DOI:** 10.1128/mbio.02544-24

**Published:** 2024-10-30

**Authors:** Erin B. Purcell

**Affiliations:** 1 Department of Chemistry and Biochemistry, Old Dominion University, Norfolk, Virginia, USA; University of Delaware, Newark, Delaware, USA

**Keywords:** *Clostridioides difficile*, oxidative stress, antioxidant, oxygen reductase

## Abstract

The obligate anaerobe *Clostridioides difficile* encodes multiple reductases to detoxify molecular oxygen and reactive oxygen species. Caulat and colleagues have characterized the activity and regulation of four such reductases (L. C. Caulat, A. Lotoux, M. C. Martins, N. Kint, et al., mBio 15:e01591-24, 2024, https://doi.org/10.1128/mbio.01591-24). Each proved critical for clostridial survival in a different range of oxygen concentrations; together, they ameliorate a broad range of oxidative stress levels. Moreover, two previously uncharacterized regulators were found to control reductase gene expression in response to oxidative stress. The genetic repressor Rex and the reductase FdpF are both sensitive to the NAP^+^:NADH ratio, which is affected by a cell’s metabolic state as well as redox activity. While oxygen is known to influence the expression of metabolism genes in *C. difficile*, the mechanisms for cross-talk between the pathways that respond to oxidative and metabolic stress are not well known. The NADH dependence of Rex and FdpF may represent a newly mapped junction between these pathways.

## COMMENTARY

The intestinal pathogen *Clostridioides difficile* is an obligate anaerobe that survives exposure to environmental oxygen during transit between hosts by differentiating into metabolically dormant, oxygen-impervious spores, which only germinate into replicative, toxin-producing vegetative cells when exposed to nutrients and bile salts in the hypoxic small intestine. However, while vegetative *C. difficile* cells avoid diatomic oxygen, they cannot ignore it. Levels of oxygen in the digestive system decline across the stomach, the small intestine, and the large intestine, but it is not completely depleted even in the colon (1). The cells of the intestinal epithelium receive oxygen through the circulatory system and release small amounts of it into the mucus that coats the gastrointestinal tract (1). In addition, mammalian immune systems employ reactive oxygen species (ROS) and reactive nitrogen species as weapons, generating them in localized bursts to kill invading pathogens (2). Anaerobic bacteria must, therefore, encode and utilize antioxidants to counteract constitutive low-level oxygen exposure as well as transiently high levels of ROS.

*C. difficile* is an extremely resilient pathogen that causes infections with high recurrence rates due to its resistance to multiple classes of antibiotics and ability to evade host immune defenses. While the ability of clostridial spores to survive exposure to environmental pressures is well documented, less is known about the survival mechanisms that allow vegetative cells to persist within a host. *C. difficile* encodes superoxide dismutase, superoxide reductase, and four reductases capable of reducing diatomic oxygen *in vitro* (3, 4). Recently, Caulat and colleagues (5) have examined the regulation and *in vivo* activity of the oxygen reductases to determine how antioxidant expression and activity are calibrated to the intensity and duration of oxidative stress.

The authors have found that while each reductase catalyzes the same reaction—the iron-dependent addition of electrons and protons to convert O_2_ into H_2_O—their expression and activity are tuned to different subsets of the range of oxygen levels that *C. difficile* could reasonably encounter in its environment. The reverse rubrerythrin revRbr2 and the flavodiiron protein FdpA are expressed under the control of the “housekeeping” sigma factor σ^A^ and mediate oxygen levels likely to be encountered by unstressed *C. difficile* in a favorable environment (4, 5). RevRbr2 is important for *C. difficile* survival at the 0.1%–0.4% oxygen concentrations found in the intestinal lumen, while FdpA is important for survival at the ~1% oxygen concentration found in the intestinal mucus (4, 5). These genes, as well as those for the highly homologous revRbr1 and FdpF, are also all positively regulated by σ^B^, the alternate transcription factor that mediates the *C. difficile* response to multiple extracellular stresses (4). Both RevRbr1 and FdpF mediate the response to oxygen levels up to 4%, the level encountered by newly germinated *C. difficile* in the small intestine, while FdpF is crucial for the survival of transient exposure to the 21% oxygen levels found in air (5). FdpF can pull the electrons it uses to reduce oxygen directly from the small molecule electron donor NADH, while the others depend on still-unknown protein partners (6).

Reductase and antioxidant gene transcription is often subject to negative regulation as they require iron, a growth-limiting bacterial nutrient (2). Here, the authors have identified two additional repressors of reductase gene transcription in *C. difficile*. OseR, which could potentially sense oxygen directly through an intermolecular disulfide bond, represses all four reductase genes in the absence of oxygen (5). In addition, Rex, which responds to the intracellular ratio of oxidized NAD^+^ to reduced NADH, represses *fdpF* in the absence of oxygen (5). The most potent reductase, utilized only under extreme oxidative stress, is thus the most tightly regulated.

The NAD^+^:NADH ratio is affected by the redox state of the cell—oxygen reduction requires electrons and depletes NADH—as well as by the metabolic state of the cell—catabolism replenishes NADH, while anabolism depletes it. A metabolically active cell will experience more fluctuations in this ratio than a dormant stationary phase or starved cell. Stationary phase and starvation are known to regulate the transcription of *C. difficile* stress response genes through the nutrient-sensitive transcriptional regulator CodY. While none of these four reductases are regulated by CodY, the “everyday” reductase *fdpA* is upregulated by glucose via CcpA, a catabolite control protein that regulates central carbon and nitrogen metabolism in *C. difficile*. (7, 8) This could reflect a need for additional antioxidant activity during periods of high metabolic activity.

The authors note that several studies have identified clostridial metabolic genes whose transcription is affected by oxygen stress, although which specific genes are affected appears to vary greatly depending on the bacterial strain and the intensity and extent of the oxygen stress (5). The discovery that different reductases specialize in responding to different levels of oxygen stress lends support to the idea that the metabolic response to oxygen exposure may be similarly dose- and duration-dependent. They also point out that *revRbr2*, encoding the reductase that responds to low-level oxygen exposure, appears to be regulated differently during growth in liquid medium and on agar plates, which the authors compare to growth within a microcolony or a biofilm (5). Oxidative stress has recently been shown to stimulate increased *C. difficile* biofilm formation via the guanosine alarmone synthetase RelQ (9). Nucleotide alarmones mediate the bacterial stringent response to starvation and other stresses, suggesting another potential link between oxidative stress and nutrient (un)availability.

Adaptation to environmental stress, especially oxidative stress, is crucial for *C. difficile* persistence within a mammalian host. This organism’s defense mechanisms must be identified and understood before they can be subverted. This work provides substantial new insights into how *C. difficile* uses a dynamic regulatory network to respond efficiently to a broad spectrum of oxidative stress intensity and offers intriguing hints of how antioxidant defenses may prove to be intimately coupled with metabolism in this organism.
